# Association Between *BRAF* Mutation Status and Clinicopathological Features in Melanoma Patients in Kosova

**DOI:** 10.3390/diseases14080278

**Published:** 2026-08-04

**Authors:** Merita Hashani, Arjeta Podrimaj-Bytyqi

**Affiliations:** 1 Faculty of Medicine, University of Prishtina, 10000 Pristina, Kosovo; merita.hashani@uni-pr.edu; 2 Institute of Pathology, University Clinical Center of Kosova, 10000 Pristina, Kosovo

**Keywords:** melanoma, Breslow thickness, *BRAF* mutation, V600E, targeted therapy, molecular profiling, Kosova

## Abstract

Background/Objective: Melanoma is an aggressive skin malignancy characterized by significant molecular heterogeneity. Among the molecular alterations identified in melanoma, *BRAF* mutations represent one of the most common genetic abnormalities and play an important role in activating the MAPK signaling pathway. *BRAF* mutation status has become clinically important because of its prognostic significance and implications for targeted therapy. This study aimed to evaluate the frequency of *BRAF* mutations and their associations with demographic, histopathological, and clinicopathological characteristics in melanoma patients at the only referral center for *BRAF* testing in Kosova, the Institute of Pathology, University Clinical Center of Kosova (UCCK). Methods: This retrospective study included 127 melanoma patients. Descriptive statistics, frequency analysis, Spearman’s correlation and multivariable binary logistic regression analyses were performed to evaluate associations between *BRAF* mutation status and clinicopathological variables, including age, gender, Breslow thickness, histological type, ulceration, and anatomical localization. Results: *BRAF* mutation was identified in 76 of 127 melanoma patients (59.8%). The *BRAF* V600E/V600E2/V600D variants represented the predominant molecular subtype (75%). *BRAF*-positive melanoma was more frequently observed in younger patients and was significantly associated with increased Breslow thickness, nodular melanoma, ulceration, and trunk localization. Conclusions: *BRAF* mutations were highly prevalent in melanoma patients from Kosova and were associated with clinicopathological features of a more aggressive disease. The findings establish an important baseline for molecular epidemiology in the country and support the integration of routine *BRAF* testing into personalized melanoma management and future regional research.

## 1. Introduction

Skin cancers are the most common cancer diagnosed worldwide. Non-melanocytic skin cancers are the most frequently encountered, while melanoma, a malignant neoplasm of the skin that arises from epidermal melanocytes (specialized pigment cells), represents a major global health concern. According to GLOBOCAN 2024, melanoma is the 17th most common cancer worldwide, with an estimated 337,960 new cases, accounting for 1.6% of all new cancer diagnoses [1,2]. Reports on melanoma incidence project a continuous global increase in the coming years [3,4]. Due to its aggressiveness, poor prognosis, and late diagnosis, it remains the leading cause of skin cancer-related mortality [2].

According to the World Health Organization (WHO), overexposure to ultraviolet (UV) radiation from both natural and artificial sources remains the primary risk factor for melanoma, a danger further influenced by genetic factors like fair skin, a high number of moles, and a history of severe sunburns. Consequently, these risk factors lead to distinct clinicopathological characteristics of melanoma, which are classified by histological type, tumor thickness (Breslow depth), the presence of ulceration, and mitotic rate. Together, these pathological features serve as the primary indicators for precise disease staging and patient prognosis [5].

Over recent decades, knowledge about the biology and pathogenesis of melanoma has improved significantly [6,7]. It is known that the development and progression of melanoma are driven by molecular changes in specific genes and changes in the tumor microenvironment [8]. Tumor microenvironment alterations, such as the overexpression of matrix metalloproteinases MMP2 and MMP-9, have been described to promote the degradation of the extracellular matrix and create a favorable environment for tumor cell proliferation and growth [9], whereas frequent somatic mutations in genes such as *BRAF*, *NRAS*, *PTEN*, *TP53* and *CDKN2A* lead to abnormal activation of two major signaling pathways, MAPK and PI3K/AKT, involved in tumor growth and progression [10].

Mitogen-activated protein kinase (MAPK) is a signaling pathway that is highly activated and frequently dysregulated in melanoma [11]. The most common genetic alterations reported to contribute to abnormal MAPK signaling are *BRAF* mutations [12]. *BRAF* mutations and their involvement in melanoma pathogenesis were first described by Davies et al. [7]. Activation of the *BRAF* mutation leads to constitutive activation of the MAPK signaling pathway independent of external growth signals, resulting in uncontrolled cell proliferation, increased survival, tumor growth, and progression in melanoma [13]. The most common *BRAF* mutation is the V600E mutation, which results from a substitution of valine (V) for glutamic acid (E) at codon 600 in exon 15 of the *BRAF* gene [14].

The frequency of *BRAF* mutations in melanoma varies among different populations and clinical characteristics, histological types of the tumor, anatomical locations, and geographic regions [15]. Previous studies have reported *BRAF* mutation frequencies ranging from approximately 40% to 60% in cutaneous melanoma [16], with lower frequencies observed in acral and mucosal subtypes [17]. Identification of *BRAF* mutation status has become clinically essential because patients with activating mutations may benefit from targeted therapies [13]. Data from clinical trials in patients treated with targeted therapies using BRAF and MEK inhibitors indicate treatment effectiveness and significant improvement in clinical outcomes in advanced melanoma patients [12,18]. As a result, molecular testing for *BRAF* mutations has become an essential component of routine diagnostics for patients diagnosed with melanoma.

The global incidence rate of melanoma varies among Caucasian populations, depending on ethnicity and geographic region. The highest incidence rate evaluated by the International Agency for Research on Cancer (IARC) is reported in Australia and New Zealand with an Age-Standardized Rate (ASR) of 38.5 and 29.2 per 100,000 inhabitants per year, respectively. This is followed by Denmark with an ASR of 27.8, Norway with 26.5 and Sweden with 23.8 per 100,000 inhabitants [1]. Conversely, the Balkan Peninsula reports lower baseline incidence rates with an ASR between 3.0 and 6.0 per 100,000 inhabitants. To evaluate the national context of our research, we referenced official data from the National Institute of Public Health of Kosova (NIPHK), published in the ‘Report of Malignant Diseases for the Period January-December 2025’ by the Department of Health Statistics. This population-based registry, which integrates oncology reporting from both public and private healthcare sectors, reports that malignant neoplasms of the skin constitute 621 registered cases, representing 16.5% of the country’s total cancer cases. Within this category, 48 new cases of cutaneous melanoma were registered nationwide in 2025 [19].

However, regional and population-specific data on the prevalence and clinicopathological characteristics associated with *BRAF* mutations remain limited for the Western Balkan region. Considering the potential influence of ethnic, environmental, and epidemiological factors on *BRAF* mutation status [15], characterizing the frequency of *BRAF* mutation within individual populations provides clinically relevant insights and contributes to optimizing diagnostic and therapeutic approaches. Therefore, this study aimed to assess the *BRAF* mutation status in melanoma patients diagnosed at the Institute of Pathology in Pristina, University Clinical Center of Kosova (UCCK), and analyze their association with clinicopathological characteristics.

## 2. Materials and Methods

### 2.1. Study Design and Patients

This study was designed as a retrospective observational study in melanoma patients at the only referral center for BRAF testing in Kosova, the Institute of Pathology, University Clinical Center of Kosova (UCCK). This study aimed to evaluate the demographic, histopathological, and molecular characteristics of patients diagnosed with melanoma, with particular focus on *BRAF* mutation status. A total of 138 melanoma patients who were tested for *BRAF* status in our Institute between September 2023 and December 2025 were initially included in the study. Among them, 11 patients were excluded because they did not meet the inclusion criteria due to insufficient tumor tissue samples (n = 9) and lack of information (n = 2). Consequently, the final evaluable study population consisted of 127 patients, a highly representative cohort, comprising the significant majority of melanoma patients during this timeframe. Clinical and pathological data were collected from patient records and histopathological reports.

### 2.2. Histopathological Evaluation

Histopathological parameters included histological types, Breslow thickness, ulceration status, and anatomical localization of the tumor. Melanoma histological types were classified as nodular melanoma, superficial spreading melanoma, mucosal melanoma, acral lentiginous melanoma, nevoid melanoma, metastatic melanoma, uveal/conjunctival melanoma, and melanoma not otherwise specified (NOS).

Breslow thickness was categorized into ≤1 mm, 1.01–2 mm, 2.01–4 mm, and >4 mm. Ulceration was evaluated based on routine histopathological examination.

### 2.3. Molecular Analysis

Molecular analysis of *BRAF* mutation status was performed using the Idylla™ Biocartis system on formalin-fixed paraffin-embedded (FFPE) (Biognost d.o.o, Zagreb, Croatia) melanoma tissue samples. The Idylla™ platform (Biocartis NV, Mechelen, Belgium) with instrument software version 28.0 and console software version 4.5.0.764. is a fully automated real-time PCR-based molecular diagnostic system that enables direct analysis of FFPE tissue sections without the need for prior DNA extraction. The assay was performed according to the manufacturer’s instructions.

Clinically relevant *BRAF* mutations analyzed included V600E/V600E2/V600D and V600K/V600R/V600M variants. Idylla *BRAF* testing results are grouped in the above-mentioned variants. Patients were categorized as *BRAF*-positive, *BRAF*-negative, or insufficient DNA when molecular analysis could not be completed due to inadequate tumor material or invalid test results.

### 2.4. Statistical Analysis

Collected data were processed and analyzed using the statistical software SPSS V.28 (IBM Corp., Armonk, NY, USA). Descriptive statistics were used to summarize demographic and clinicopathological characteristics. Frequency distribution and percentage analysis were applied for categorical variables.

Multivariable binary logistic regression analysis was performed to identify factors independently associated with *BRAF* mutation positivity. Spearman’s correlation analysis was used to evaluate correlations between *BRAF* mutation status and clinicopathological variables. A *p*-value < 0.05 was considered statistically significant throughout the study.

## 3. Results

Among the 127 melanoma patients included in the study, 66.9% were male and 33.1% were female. The most represented age group was ≥70 years (36.2%), followed by the 60–69 age group (21.3%).

Histological types of melanomas are considered important clinicopathological parameters for determining their aggressiveness and biological behaviors. Therefore, we analyzed the melanoma cohort based on the histopathological diagnosis. The nodular melanoma was the most frequent histological type, identified in 58 cases (45.7%). Metastatic melanoma was observed in 24 cases (18.9%), while melanoma not otherwise specified (NOS) was observed in 21 cases (16.5%). Superficial spreading melanoma was identified in 9 patients (7.1%), whereas less frequent types included mucosal melanoma, uveal/conjunctival melanoma, and acral lentiginous melanoma (Table 1).

Ulceration is an important histopathological and prognostic parameter associated with tumor aggressiveness and patient outcome. Ulceration is included in the AJCC melanoma staging system and is routinely evaluated in pathological reports. Ulceration was present in 55 cases (43.3%), suggesting a considerable proportion of tumors with more aggressive biological behavior. Ulceration was absent in 43 cases [33.9%], while data was not available in 29 cases (22.8%) (Table 1). These are the cases of metastatic melanoma and other types where ulceration is not assessable.

The results indicate that melanoma was more frequent among older patients and males, nodular melanoma was the predominant histopathological type, and there was a relatively high frequency of ulceration, reflecting histopathological characteristics associated with more aggressive disease progression.

### 3.1. BRAF Mutation Analysis

We analyzed 127 formalin-fixed, paraffin-embedded tissue blocks mainly taken from primary or metastatic sites to determine the mutational status of *BRAF*. The analysis performed with the Idylla platform showed positive *BRAF* mutations in 76 melanoma patients (59.8%). The mutation subtype V600E/V600E2/V600D was detected in 57 patients (44.8%), whereas V600K/V600R/V600M mutations were detected in 19 cases (15%). Fifty-one patients (40.2%) were negative for *BRAF* mutation or wild type (Table 2).

The molecular analysis of melanoma patients demonstrated that most positive cases showed mutations in V600E/V600E2/V600D. These variants were identified in 57 out of the 76 *BRAF*-positive cases (75%), representing the dominant molecular subtype of the mutation (Table 2). Meanwhile, V600K/V600R/V600M mutations were detected in 19 cases (25.0%). Although these variants are less frequent compared to V600E/V600E2/V600D, they remain clinically relevant for the selection of targeted therapy.

The results demonstrate a high prevalence of *BRAF* mutations in melanoma patients, particularly the V600E/V600E2/V600D variants, which are considered the most common and clinically significant mutations for targeted therapy.

### 3.2. Association Between BRAF Mutation Status and Demographic Characteristics of Cohort

The analysis of the association between *BRAF* mutation status and patient gender demonstrated that positive *BRAF* mutation was present in both genders, although with a slightly higher frequency among males (Table 3). Among 85 male patients, 53 cases (62.3%) were *BRAF*-positive, while 32 cases (37.6%) were *BRAF*-negative. Among female patients, 23 out of 42 cases (54.7%) were positive for *BRAF* mutation, whereas 19 cases (45.2%) were negative. Although the difference between genders was not pronounced, the results suggest a slightly higher prevalence of *BRAF* mutations among males.

The data indicate that *BRAF* mutation is similarly distributed between males and females, suggesting that gender may not have a major influence on the presence of *BRAF* mutation in melanoma patients.

Next, we analyzed the association between *BRAF* mutation status and patient age (Table 3). Patients younger than 40 years showed *BRAF* positivity in 73.3% of cases. In the 40–49 age group, about 66.7% of cases were *BRAF*-positive. In the 50–59 age group, *BRAF* positivity was 62.5%, while in the 60–69 age group, it decreased to 55.6%. *BRAF* positivity observed in patients aged ≥70 years was 54.3%.

The classic *BRAF* mutations V600E/V600E2/V600D were more often encountered at younger ages, whereas the mutations V600K/V600R/V600M were significantly associated with older age.

The results suggest an association between younger age and a higher prevalence of *BRAF* mutation, supporting findings from previous studies indicating that *BRAF* mutations are more common in younger individuals.

### 3.3. Association Between BRAF Status and Histopathological Features

Next, the association between *BRAF* mutation status and histological type was analyzed to investigate whether certain melanoma types are more commonly associated with *BRAF* mutations (Table 4). The positive *BRAF* mutation status was more frequent in nodular melanoma, where 39 of 58 cases (67.2%) were positive. Metastatic melanoma also showed a high rate of *BRAF* positivity, with 16 positive cases out of 24 total cases (66.7%). Melanoma NOS and superficial spreading melanoma showed a relatively balanced distribution between *BRAF*-positive and *BRAF*-negative cases. In contrast, nevoid melanoma demonstrated a higher prevalence of *BRAF* mutation, with 75.0% of cases being positive. No positive *BRAF* mutations were identified in mucosal melanoma. One case of acral lentiginous melanoma showed *BRAF*-positive status. Uveal/conjunctival melanoma demonstrated an equal distribution between positive and negative cases.

These findings suggest that *BRAF* mutation is more commonly associated with nodular and metastatic forms of melanoma, while acral and mucosal melanomas demonstrate lower frequencies of *BRAF* positivity.

Next, the association between Breslow thickness and *BRAF* mutation status is analyzed (Table 4). Among melanomas with Breslow thickness ≤ 1 mm, 57.1% of cases were *BRAF*-positive. In the 1.01–2 mm category, 58.3% were *BRAF*-positive. In the 2.01–4 mm category, 65.3% of patients were positive for *BRAF* mutation. The highest frequency of *BRAF* mutation was observed in patients with Breslow thickness > 4 mm, where 32 out of 44 cases (72.7%) were *BRAF*-positive. Cases categorized as “not determined” included patients in whom Breslow thickness is not applicable, such as in metastatic cases or certain melanoma types.

These findings suggest a possible association between *BRAF* mutation and melanomas with greater tumor thickness, which are generally linked to more aggressive disease progression and poorer prognosis. Overall, the data supports the prognostic significance of Breslow thickness and suggests that *BRAF* mutation may be more frequent in melanomas with more advanced invasion.

To evaluate the potential differences in molecular characteristics according to tumor site, the association between *BRAF* mutation and anatomical location was analyzed (Table 4). The positive *BRAF* mutation was more frequent in melanomas located on the trunk, where 23 out of 32 cases (71.9%) were positive. Melanomas of the lower extremities showed a *BRAF* positivity rate of 62.1%, while melanomas of the upper extremities demonstrated 57.9% positivity. In melanomas localized to the head and neck region, *BRAF*-positive cases accounted for about 52.0% of all cases. Mucosal melanomas showed no cases with positive *BRAF* mutation, whereas ocular/conjunctival melanomas demonstrated an equal distribution between positive and negative cases.

The results suggest that *BRAF* mutations are more frequently associated with cutaneous melanomas of the trunk and extremities, reflecting distinct molecular profiles according to the anatomical localization of the tumor.

### 3.4. Association Between Clinicopathological Factors and BRAF Mutation Status

Multivariable binary logistic regression and Spearman’s correlation analyses were performed to evaluate factors associated with *BRAF* mutation status in melanoma patients (Table 5). In the multivariable logistic regression analysis, *BRAF* mutation status was considered the dependent variable, while age (≥60 years), Breslow thickness (>4 mm), histological type (nodular melanoma), ulceration status, anatomical localization (trunk), and gender were included as independent variables. Adjusted odds ratios (ORs) with 95% confidence intervals (CIs) were calculated.

The multivariable binary logistic regression analysis demonstrated that patients aged ≥60 years had lower odds of harboring *BRAF* mutation compared with younger patients (adjusted OR = 0.58; 95% CI: 0.31–0.91; *p* = 0.032). Greater Breslow thickness was significantly associated with *BRAF*-positive status, with patients with a Breslow thickness > 4 mm showing higher odds of *BRAF* mutation (adjusted OR = 2.11; 95% CI: 1.18–3.76; *p* = 0.014). Nodular melanoma was also independently associated with increased odds of *BRAF* positivity (adjusted OR = 1.89; 95% CI: 1.02–3.44; *p* = 0.041). Similarly, ulceration presence (adjusted OR = 1.76; 95% CI: 1.01–3.08; *p* = 0.047) and trunk localization (adjusted OR = 2.24; 95% CI: 1.19–4.01; *p* = 0.018) were significantly associated with higher odds of *BRAF* mutation. Gender was not significantly associated with *BRAF* mutation status (adjusted OR = 1.21; 95% CI: 0.74–2.08; *p* = 0.284).

Spearman’s correlation analysis showed a significant negative correlation between age and *BRAF* mutation status (r_s_ = −0.284; *p* = 0.011), indicating a higher frequency of *BRAF* mutations among younger patients. Positive correlations were observed between *BRAF* mutation status and Breslow thickness (r_s_ = 0.318; *p* = 0.004), nodular melanoma (r_s_ = 0.267; *p* = 0.018), ulceration (r_s_ = 0.241; *p* = 0.026), and trunk localization (r_s_ = 0.296; *p* = 0.009). No statistically significant correlation was observed between gender and *BRAF* mutation status (r_s_ = 0.087; *p* = 0.287).

Overall, both multivariable binary logistic regression and Spearman’s correlation analyses demonstrated consistent associations between *BRAF* mutation status and key clinicopathological characteristics, including younger age, increased Breslow thickness, nodular melanoma type, ulceration, and trunk localization.

## 4. Discussion

The present study analyzed the demographic, histopathological, and molecular characteristics associated with *BRAF* mutation status in a highly representative group of melanoma patients from Kosova. Our findings demonstrate a high prevalence of *BRAF* mutations, with V600E/V600E2/V600D mutations representing the dominant molecular subtype. These results are consistent with previous studies reporting V600E *BRAF* mutation as the most common genetic alteration in cutaneous melanoma [7,13].

The classic V600E/V600E2/V600D mutation was found in up to 75% of cases, followed by the non-classical V600K/V600R/V600M subtype found in 25% of cases. Interestingly, our cohort study showed a high rate of non-classical V600K/V600R/V600M mutation subtype in the older age group. This mutation is reported to show variation in different populations [20], which might be related to some environmental and geographic properties as well as UV exposure [21]. Further investigation is needed to determine the relationship between environmental factors, the frequency rate, and the *BRAF* mutation subtype in different geographical regions.

In our study, *BRAF*-positive melanoma was more common in younger patients. The prevalence of *BRAF* mutation gradually decreased with increasing age, supporting previous evidence that *BRAF*-mutated melanoma is associated with earlier age at diagnosis [22]. Younger patients may develop melanomas through molecular pathways associated with intermittent ultraviolet (UV) exposure and increased activation of the MAPK signaling pathway, which plays a central role in melanoma tumorigenesis [11,23].

The current cohort also demonstrated an association between *BRAF* mutation and greater Breslow thickness. Melanomas with Breslow thickness > 4 mm showed the highest percentage of *BRAF*-positive cases. Increased Breslow thickness is a well-established negative prognostic factor associated with deeper tumor invasion, increased metastatic potential, and reduced survival [23,24]. Similar associations between *BRAF* mutation and increased tumor thickness have been reported in previous studies, suggesting that *BRAF*-driven melanomas may exhibit more aggressive biological behavior and enhanced proliferative activity [25,26]. The observed association in the present study further supports the hypothesis that *BRAF* mutation may contribute to melanoma progression and tumor aggressiveness.

Similarly, ulceration demonstrated a positive association with *BRAF* mutation status. Ulceration is recognized as an indicator of aggressive tumor biology and is incorporated into melanoma staging systems because of its prognostic significance [2,27]. Previous studies have also reported a higher frequency of ulceration in *BRAF*-mutated melanoma, suggesting that MAPK pathway dysregulation may contribute to increased tumor proliferation and more aggressive clinicopathological behavior [26,27]. The higher prevalence of ulceration among *BRAF*-positive melanomas observed in the present study may therefore reflect enhanced tumor growth and biological aggressiveness.

Regarding histological type, nodular melanoma demonstrated the strongest association with *BRAF* positivity. Nodular melanoma is generally characterized by rapid vertical growth, increased Breslow thickness, and poorer clinical prognosis compared with other melanoma histological types [28]. Previous molecular studies have also demonstrated a high prevalence of *BRAF* mutations in nodular melanoma, supporting the concept that enhanced tumor progression in cutaneous melanoma is *BRAF*-driven [27]. The predominance of *BRAF* mutations in nodular melanoma observed in the present study further supports this association.

Anatomical localization was another clinicopathologic feature correlated with *BRAF* mutation status, as this mutation was found to be more frequently identified in melanomas located on the trunk and extremities. These findings suggest that intermittently sun-exposed skin is more likely to harbor *BRAF* mutations, a finding consistent with previous studies. Within our cohort, mucosal and acral melanomas showed lower frequencies of *BRAF* positivity. However, our subgroup sample size for these specific subtypes remains limited; therefore, a larger cohort is required to definitively validate these lower mutation rates. Mucosal and acral melanomas exhibit other alternative molecular alterations, including *KIT* and *NRAS* mutations [6,8]. Recent genomic studies have further confirmed the distinct molecular profiles of acral and mucosal melanoma compared with cutaneous melanoma [18].

No significant association was found between gender and *BRAF* mutation status. Although male patients demonstrated slightly higher *BRAF* positivity rates, the difference was not statistically significant. Though some studies indicated modest gender-related differences in melanoma biology [14], patient gender does not represent an independent predictor of *BRAF* mutation in melanoma patients [29].

The statistical analysis, including tests such as multivariable binary logistic regression analysis and Spearman’s correlation analyses, consistently supported the association of *BRAF* mutation with younger age, increased Breslow thickness, nodular subtype, ulceration, and trunk localization. Our data aligns with previous reports evaluating the prognostic and molecular significance of *BRAF* mutation [13,15,30]. The concordance between different statistical approaches in the present study strengthens the reliability of the observed associations and further supports the role of *BRAF* mutation as a marker associated with distinct and potentially more aggressive melanoma behavior [26].

The clinical importance of these findings lies in the role of *BRAF* mutation testing in personalized melanoma management. Identification of *BRAF*-mutated melanoma is essential because patients harboring activating *BRAF* mutations may benefit from targeted therapy using BRAF and MEK inhibitors, which have significantly improved survival outcomes in advanced melanoma [31]. Combination therapies targeting the MAPK signaling pathway have demonstrated substantial improvements in progression-free survival and overall survival compared with conventional chemotherapy [18]. These findings highlight the importance of routine molecular profiling for therapeutic decision-making and individualized melanoma treatment strategies.

Overall, the present study confirms the high prevalence of *BRAF* mutations in melanoma patients and demonstrates significant associations between *BRAF* positivity and aggressive clinicopathological characteristics. These findings further support the importance of routine molecular profiling in melanoma diagnosis, prognostic assessment, and targeted therapeutic decision-making, particularly in the era of personalized melanoma treatment strategies.

Novelty of the study:

The novelty of this study lies in providing the first comprehensive assessment of BRAF mutation status and its association with clinicopathological characteristics in melanoma patients from Kosova, an underrepresented population with limited molecular epidemiological data. By evaluating a highly representative cohort of 127 patients tracked from 2023 to 2025, this study comprises the majority of melanoma cases during this timeframe, offering highly reliable data for Kosova. Our findings provide valuable regional evidence that may support future melanoma research and the implementation of precision oncology in Kosova.

Limitations of the study:

The main limitations of the study are the relatively small number of melanoma cases, reflecting Kosova’s small and young population, and the single-center design, with all cases originating from the country referral pathology center. While we used multivariable binary logistic regression to adjust for clinical heterogeneity (tumor site and histology), the mixed cohort composition means our analyses should be interpreted as exploratory baseline data requiring future validation in larger, multicenter international registries. Despite these limitations, the study provides valuable baseline molecular epidemiological data for Kosova, representing the large majority of national melanoma patients during this timeframe.

## 5. Conclusions

In conclusion, our results demonstrate a high *BRAF* mutation frequency among melanoma patients in Kosova. The predominant molecular subtype was found to be V600E/V600E2/V600D. Younger age, increased Breslow thickness, nodular melanoma, ulceration, and trunk localization were significantly associated with *BRAF* positivity. These findings suggest that *BRAF*-mutated melanoma may be associated with more aggressive tumor behavior and distinct biological characteristics.

This study is the first of its kind to show the molecular profile of melanoma patients in Kosova and confirms the association between clinicopathological features and *BRAF* status. These results contribute to the global body of data regarding melanoma research. Further large-scale multicenter studies are recommended to validate these associations and to better understand the prognostic and therapeutic implications of *BRAF* mutation across different melanoma subtypes.

Overall, the results support the integration of routine *BRAF* mutation testing into melanoma diagnosis, prognostic evaluation, and personalized therapeutic decision-making. Our data provides important regional insights first and foremost for clinicians in Kosova, supporting the clinical utility of *BRAF* mutation analysis in melanoma management.

## Figures and Tables

**Table 1:** Demographic and histopathologic characteristics of patients.

Variable	Category	n	%
Gender	Male	85	66.9
	Female	42	33.1
Age Group [years]	<40	15	11.8
	40–49	15	11.8
	50–59	24	18.9
	60–69	27	21.3
	≥70	46	36.2
Histological type	Nodular melanoma	58	45.7
	Metastatic melanoma	24	18.9
	Melanoma NOS	21	16.5
	Superficial spreading melanoma	9	7.1
	Acral lentiginous melanoma	6	4.7
	Nevoid melanoma	4	3.1
	Mucosal melanoma	3	2.4
	Uveal/Conjunctival melanoma	2	1.6
Ulceration	Present	55	43.3
	Absent	43	33.9
	Not determined	29	22.8

**Table 2:** Frequency of *BRAF* mutation status and molecular subtype.

*BRAF* Mutation Status	n	%	*BRAF* Mutation Subtype %
V600E/V600E2/V600D	57	44.8	75.0
V600K/V600R/V600M	19	15	25.0
*BRAF* Negative	51	40.2	
Total	127	100.0	100.0

**Table 3:** Association between *BRAF* status, gender, and age in melanoma patients.

Gender	*BRAF*-Negative n [%]	*BRAF*-Positive n [%]	Total
Male	32 [37.6]	53 [62.3]	85
Female	19 [45.2]	23 [54.7]	42
**Age Group [years]**	***BRAF*-Negative n [%]**	***BRAF*-Positive n [%]**	**Total**
<40	4 [26.7]	11 [73.3]	15
40–49	5 [33.3]	10 [66.7]	15
50–59	9 [37.5]	15 [62.5]	24
60–69	12 [44.4]	15 [55.6]	27
≥70	21 [45.7]	25 [54.3]	46

**Table 4:** Association between *BRAF* status and histopathological features.

Histological Type	*BRAF*-Negative n [%]	*BRAF*-Positive n [%]	Total
Nodular melanoma	19 [32.8]	39 [67.2]	58
Metastatic melanoma	8 [33.3]	16 [66.7]	24
Melanoma NOS	11 [52.4]	10 [47.6]	21
Superficial spreading melanoma	4 [44.4]	5 [55.6]	9
Acral lentiginous melanoma	5 [83.3]	1 [16.7]	6
Nevoid melanoma	1 [25.0]	3 [75.0]	4
Mucosal melanoma	3 [100.0]	0 [0.0]	3
Uveal/Conjunctival melanoma	1 [50.0]	1 [50.0]	2
**Breslow Thickness**	***BRAF*-Negative n [%]**	***BRAF*-Positive n [%]**	**Total**
≤1 mm	3 [42.9]	4 [57.1]	7
1.01–2 mm	10 [41.7]	14 [58.3]	24
2.01–4 mm	8 [34.7]	15 [65.3]	23
>4 mm	12 [27.3]	32 [72.7]	44
Not determined	18 [62.0]	11 [38.0]	29
**Anatomical Localization**	***BRAF*** **-Negative n [%]**	***BRAF*** **-Positive n [%]**	**Total**
Trunk	9 [28.1]	23 [71.9]	32
Lower extremities	11 [37.9]	18 [62.1]	29
Upper extremities	8 [42.1]	11 [57.9]	19
Head and neck	12 [48.0]	13 [52.0]	25
Mucosal	3 [100.0]	0 [0.0]	3
Ocular/Conjunctival	1 [50.0]	1 [50.0]	2
Skin, not specified	7 [41.2]	10 [58.8]	17

**Table 5:** Multivariable binary logistic regression and Spearman’s correlation analyses of factors associated with *BRAF* mutation status in melanoma patients.

Variable	OR	95% CI	*p*-Value	Spearman’s r_s_	*p*-Value
Age ≥ 60 years	0.58	0.31–0.91	0.032 *	−0.284	0.011 *
Breslow thickness > 4 mm	2.11	1.18–3.76	0.014 *	0.318	0.004 *
Nodular melanoma	1.89	1.02–3.44	0.041 *	0.267	0.018 *
Ulceration present	1.76	1.01–3.08	0.047 *	0.241	0.026 *
Trunk localization	2.24	1.19–4.01	0.018 *	0.296	0.009 *
Gender	1.21	0.74–2.08	0.284	0.087	0.287

* Statistically significant association/correlation (*p* < 0.05). Adjusted ORs were obtained from the multivariable binary logistic regression model. Spearman’s correlation coefficient (r_s_) was used to assess the correlation between *BRAF* mutation status and clinicopathological variables.

## Data Availability

The materials supporting the data presented in this study are available upon request.
